# State-Level Patterns and Trends in Cigarette Smoking Across Racial and Ethnic Groups in the United States, 2011–2018

**DOI:** 10.5888/pcd18.200507

**Published:** 2021-05-06

**Authors:** Sarah D. Mills, Yajing Hao, Alison M. Elliott, Christopher A. Wiesen

**Affiliations:** 1 Department of Health Behavior, Gillings School of Global Public Health, University of North Carolina, Chapel Hill, Chapel Hill, North Carolina; 2 Lineberger Comprehensive Cancer Center, University of North Carolina, Chapel Hill, Chapel Hill, North Carolina; 3 Department of Biostatistics, Gillings School of Global Public Health, University of North Carolina, Chapel Hill, Chapel Hill, North Carolina; 4 Odum Institute, University of North Carolina, Chapel Hill, Chapel Hill, North Carolina

## Abstract

**Introduction:**

Reducing racial/ethnic disparities in smoking is a priority for state tobacco control programs. We investigated disparities in cigarette use by race/ethnicity, as well as trends in cigarette use across racial/ethnic groups from 2011 to 2018 in 50 US states and the District of Columbia.

**Methods:**

We used data from the Behavioral Risk Factor Surveillance System. In each state, smoking prevalence and corresponding 95% CIs were estimated for each racial/ethnic group in 2011, 2014, and 2018. We used logistic regression models to examine state-specific linear and quadratic time trends in smoking prevalence from 2011 to 2018.

**Results:**

Racial/ethnic disparities in smoking prevalence varied across states. From 2011 to 2018, compared with White adults, the odds of smoking were lower among Black adults in 14 states (odds ratio [OR] range, 0.58–0.91) and were higher in 9 states (OR range, 1.10–1.98); no differences were found in the odds of smoking in 13 states. Compared with White adults, the odds of smoking were lower among Hispanic adults in most states (OR range, 0.33–0.84) and were typically higher among Other adults (OR range, 1.19–2.44). Significant interactions between year and race/ethnicity were found in 4 states, indicating that time trends varied across racial/ethnic groups. In states with differential time trends, the decline in the odds of smoking was typically greater among Black, Hispanic, and Other adults compared with White adults.

**Conclusion:**

Some progress in reducing racial/ethnic disparities in smoking has been made, but additional efforts are needed to eliminate racial/ethnic disparities in smoking.

**Summary**

**What is already known on this subject?**

Smoking prevalence has declined in the United States during the past several decades. However, disparities in smoking prevalence across racial/ethnic groups remain.

**What is added by this report?**

Trends in smoking prevalence varied across racial/ethnic groups in only 4 states from 2011 to 2018. In states with differential time trends, the decline in the odds of smoking was typically greater among Black, Hispanic, and Other adults compared with White adults.

**What are the implications for public health practice?**

Some progress has been made in reducing racial/ethnic disparities in smoking. Examining trends in state-level smoking prevalence across racial/ethnic groups provides insight into which demographic groups may benefit from targeted tobacco control efforts.

**MEDSCAPE CME**

In support of improving patient care, this activity has been planned and implemented by Medscape, LLC and *Preventing Chronic Disease*. Medscape, LLC is jointly accredited by the Accreditation Council for Continuing Medical Education (ACCME), the Accreditation Council for Pharmacy Education (ACPE), and the American Nurses Credentialing Center (ANCC), to provide continuing education for the healthcare team.

Medscape, LLC designates this Journal-based CME activity for a maximum of 1.00 AMA PRA Category 1 Credit(s)™. Physicians should claim only the credit commensurate with the extent of their participation in the activity.

Successful completion of this CME activity, which includes participation in the evaluation component, enables the participant to earn up to 1.0 MOC points in the American Board of Internal Medicine’s (ABIM) Maintenance of Certification (MOC) program. Participants will earn MOC points equivalent to the amount of CME credits claimed for the activity. It is the CME activity provider’s responsibility to submit participant completion information to ACCME for the purpose of granting ABIM MOC credit.

**Release date: May 6, 2021; Expiration date: May 6, 2022**

**Learning Objectives**

Upon completion of this activity, participants will be able to:

1. Assess the overall rate of smoking in the United States
2. Analyze trends in smoking rates among Black adults in the United States
3. Analyze trends in smoking rates among Hispanic adults in the United States
4. Distinguish differences in smoking rates according to race/ethnicity in different states

**EDITOR**

Camille Martin, RD

Editor

Preventing Chronic Disease 

Disclosure: Camille Martin has disclosed no relevant financial relationships.

**CME AUTHOR**

Charles P. Vega, MD

Health Sciences Clinical Professor of Family Medicine

University of California, Irvine School of Medicine

Disclosure: Charles P. Vega, MD, has disclosed the following relevant financial relationships: Served as an advisor or consultant for: GlaxoSmithKline

**AUTHORS **

Sarah D. Mills, PhD, MPH

Department of Health Behavior

Gillings School of Global Public Health

Lineberger Comprehensive Cancer Center

University of North Carolina, Chapel Hill

Disclosure: Sarah D. Mills, PhD, MPH, has disclosed no relevant financial relationships.

Yajing Hao

Department of Biostatistics

Gillings School of Global Public Health

University of North Carolina, Chapel Hill

Disclosure: Yajing Hao has disclosed no relevant financial relationships.

Alison M. Elliott

Department of Health Behavior

Gillings School of Global Public Health

University of North Carolina, Chapel Hill

Disclosure: Alison M. Elliott, has disclosed no relevant financial relationships.

Christopher A. Wiesen, PhD

Odum Institute

University of North Carolina, Chapel Hill

Disclosure: Christopher A. Wiesen, PhD, has disclosed no relevant financial relationships.

## Introduction

Eliminating disparities in smoking across racial/ethnic groups is a priority for tobacco control because it is critical to reducing overall smoking prevalence in the United States. Despite declines in smoking at the national level, disparities remain across racial/ethnic groups (1–3). In 2018, 13.7% of adults reported smoking (1). Smoking prevalence was higher than the nationwide prevalence among American Indian/Alaska Native, White, and Black adults (1), and prevalence was lower among Hispanic and Asian adults (1).

Examining trends in smoking may provide insight into current racial/ethnic disparities. At the national level, research suggests that smoking prevalence is not declining at the same rate across racial/ethnic groups (4–6). Asian adults had the lowest smoking prevalence in 2002 and the greatest relative percentage change in smoking from 2002 to 2016, with a 53% reduction in smoking prevalence (6). The relative percentage change was between 34% and 37% among Native Hawaiian/Other Pacific Islander and Hispanic adults and between 21% and 24% among White and Black adults (6). Despite having the highest smoking prevalence in 2002, the relative percentage change among multiracial adults was only 17%. There was no significant change in smoking prevalence among American Indian/Alaska Native adults (6).

Smoking prevalence and trends across racial/ethnic groups provide critical information at the national level, but differences across states may be obscured. State tobacco control programs have the authority to implement tobacco control policies (7), but state policies vary widely, which may result in variation in racial/ethnic disparities in smoking across states (7,8). 

Therefore, we investigated disparities in cigarette use by race/ethnicity, as well as trends in cigarette use across racial/ethnic groups from 2011 to 2018 in 50 US states and the District of Columbia. Our study is the first to examine recent state-level trends in racial/ethnic disparities in smoking prevalence. Examining trends in state-level smoking prevalence may help identify which states are making progress toward health equity.

## Methods

Data for this study come from the Behavioral Risk Factor Surveillance System (BRFSS). The BRFSS is a state-representative, random digit–dialed telephone survey that collects data annually about health-related risk behaviors and health conditions among noninstitutionalized adults (aged ≥18 y) living in the United States and participating territories (9). The survey is conducted in all 50 US states; the District of Columbia; Guam; Puerto Rico; and the US Virgin Islands. Our study was limited to data collected in the core survey from 2011 to 2018 in the 50 US states and the District of Columbia (hereinafter referred to as “states”). The median landline response rate from 2011 to 2018 ranged from 45% to 53%, and the median cellular telephone response rate ranged from 28% to 47%.

The core survey of the BRFSS includes questions about adults’ smoking status and demographic characteristics. From 2011 to 2018, more than 400,000 adults completed the BRFSS each year. For each state and year, sample sizes ranged from 2,758 to 36,955 adults. A detailed description of BRFSS methods is available at www.cdc.gov/brfss/index.html.

### Measures

The following 2 questions were used to determine respondents’ smoking status: “Have you smoked at least 100 cigarettes in your entire life?” and “Do you now smoke cigarettes every day, some days, or not at all?” A respondent was considered to be a current smoker if they had smoked at least 100 cigarettes in their lifetime and smoke every day or some days.

The following demographic characteristics were assessed and categorized in the following manner for analysis: age in years (18–24, 25–34, 35–44, 45–54, 55–64, ≥65); sex (male, female); education (less than a college or technical school graduate, graduated from college/technical school or higher); and race/ethnicity (non-Hispanic White [White], non-Hispanic Black [Black], non-Hispanic multiracial or non-Hispanic “Other” [Other], Hispanic [Hispanic]).

### Data analysis

We examined descriptive statistics for the total sample (2011 through 2018). In each state, smoking prevalence and corresponding 95% CIs were estimated for each racial/ethnic group (Black, Hispanic, Other, White) in 2011, 2014, and 2018, and unadjusted time trends (linear and quadratic) in smoking were examined from 2011 to 2018 (more information available at https://tarheels.live/pcdsupplementalfile). We also used adjusted logistic regression models to examine state-specific linear and quadratic time trends in smoking prevalence from 2011 to 2018 (inclusive). Specifically, logistic regression models were estimated to examine the relationship between year (2011–2018), year-squared, and cigarette smoking status (1 = current smoker, 0 = noncurrent smoker), adjusting for age, sex, race/ethnicity, and education. If the quadratic time trend was not significant (*P* < .05), it was removed from the logistic regression model and only the linear time trend was included.

Next, logistic regression models that also included an interaction term between year and race/ethnicity were estimated to examine whether differential time trends in smoking existed between racial/ethnic groups. If the quadratic time trend was significant in the initial logistic regression model, an interaction term between year-squared and race/ethnicity was also included. If there was a significant interaction term between year and race/ethnicity or year-squared and race/ethnicity, simple effects tests were used to estimate the time trend separately in each racial/ethnic group. Adjusted smoking prevalence estimates were obtained in each year from 2011 to 2018 for racial/ethnic groups with differential time trends.

Analyses were conducted in SAS version 9.4 (SAS Institute, Inc) using recommended procedures to account for the complex survey design of the BRFSS data, including the use of statistical procedures for stratification, clustering, and sample weights (10). We also followed recommended guidelines from the Centers for Disease Control and Prevention to assess the reliability of smoking prevalence estimates by examining the total number of respondents that contributed to the denominator of the estimate and by examining the relative standard error of the estimate (11). We calculated the relative standard error by dividing the standard error by the estimate and multiplying by 100. Smoking prevalence estimates were suppressed if they were based on fewer than 50 respondents in the denominator or if the estimate had a relative standard error greater than 30% (11).

## Results

Sociodemographic characteristics for the study sample are provided in Table 1, and smoking prevalence in 2011, 2014, and 2018 is presented for each racial/ethnic group in Table 2.

**Table 1:** Demographic Characteristics of Sample, Behavioral Risk Factor Surveillance System (BRFSS), 2011–2018^a^

Demographic Characteristic	Unweighted Sample Size, No.	Weighted Sample Size, No.	Percentage of Total Sample
**Age, y**			
18–24	202,430	254,808,033	12.7
25–34	376,249	346,022,123	17.3
35–44	450,853	331,211,730	16.6
45–54	624,144	349,285,909	17.5
55–64	829,854	32,7431,931	16.4
≥65	1,270,272	386,041,090	19.4
**Sex**			
Female	2,173,864	1,023,919,498	51.4
Male	1,578,475	970,082,877	48.7
Missing	1,463	—	—
**Education**			
Less than college/technical school graduate	2,392,837	1,460,440,334	73.6
Graduated from college/technical school or higher	1,344,201	523,874,894	26.4
Missing	16,764	—	—
**Race/ethnicity**			
Non-Hispanic White	2,853,543	1,252,200,151	63.9
Non-Hispanic Black	300,514	228,988,937	11.7
Hispanic	296,518	324,414,918	16.5
Non-Hispanic Other^b^	241,001	155,228,739	7.9
Missing	62,226	—	—
**Year**			
2011	506,467	238,011,292	11.9
2012	475,687	243,057,710	12.2
2013	491,773	246,024,416	12.3
2014	464,664	248,482,532	12.5
2015	441,456	251,347,138	12.6
2016	486,303	254,151,136	12.7
2017	450,016	255,653,205	12.8
2018	437,436	258,073,387	12.9

Abbreviation: — , not applicable.

a Descriptive statistics are for combined BRFSS data from 2011 to 2018.

b Other race/ethnicity includes non-Hispanic multiracial and non-Hispanic “Other” adults.

**Table 2:** Prevalence of Cigarette Use Across Racial/Ethnic Groups, Behavioral Risk Factor Surveillance System, 2011, 2014, and 2018^a^

States	Black			Hispanic			Other			White		
	2011	2014	2018	2011	2014	2018	2011	2014	2018	2011	2014	2018
Alabama	20.8 (18.0–23.5)	20.2 (17.5–22.9)	18.5 (16.0 21.1)	27.4 (14.7–40.2)	—	10.6 (3.8–17.3)	37.2 (27.8–46.6)	16.1 (10.0–22.1)	31.3 (22.3–40.3)	25.0 (23.2–26.7)	21.9 (20.3– 23.5)	19.2 (17.6–20.8)
Alaska	—	—	—	22.9 (11.6–34.1)	12.2 (5.6–18.8)	—	31.9 (27.1–36.6)	29.7 (25.4–34.1)	31.6 (26.0–37.2)	19.3 (17.2–21.4)	17.6 (15.7–19.5)	14.8 (12.6–16.9)
Arizona	27.3 (16.5–38.1)	16.2 (10.9–21.6)	12.3 (6.3–18.3)	13.3 (9.7–17.0)	14.0 (11.5–16.5)	13.3 (10.4–16.1)	18.0 (11.2–24.8)	16.3 (12.3–20.3)	14.9 (10.1–19.8)	21.7 (19.1–24.3)	17.6 (16.3–19.0)	14.2 (12.7–15.7)
Arkansas	27.3 (20.4–34.2)	28.9 (23.1–34.7)	21.0 (16.2–25.8)	—	—	15.8 (6.8–24.7)	31.6 (19.4–43.7)	27.7 (18.7–36.8)	33.4 (22.7–44.1)	27.4 (25.1–29.8)	24.5 (22.3–26.8)	23.1 (21.1–25.1)
California	19.8 (15.6–23.9)	23.2 (18.1–28.2)	13.5 (9.5–17.5)	12.0 (10.7–13.4)	11.4 (9.7–13.1)	11.3 (10.0–12.6)	10.9 (9.0–12.9)	10.5 (8.0–13.0)	9.1 (7.2–11.1)	15.1 (14.1–16.1)	13.7 (12.4–15.0)	11.7 (10.5–12.9)
Colorado	23.2 (15.9–30.4)	22.8 (17.0–28.7)	20.6 (13.3–27.9)	20.2 (17.1–23.4)	17.4 (14.9–19.8)	16.1 (13.8–18.3)	17.0 (12.2–21.8)	15.9 (11.9–19.9)	19.8 (15.0–24.5)	17.6 (16.4–18.7)	14.8 (13.8–15.8)	13.4 (12.3–14.5)
Connecticut	20.8 (15.8–25.7)	18.5 (14.0–22.9)	18.2 (14.6–21.9)	17.1 (12.9–21.2)	20.6 (16.3–24.8)	16.5 (13.2–19.9)	16.4 (11.5–21.3)	16.0 (10.5–21.5)	8.7 (5.8–11.6)	16.8 (15.2–18.5)	14.1 (12.7–15.5)	10.8 (9.8–11.8)
Delaware	21.5 (16.8–26.3)	17.1 (12.7–21.5)	17.4 (13.6–21.2)	26.6 (17.7–35.5)	10.1 (5.8–14.4)	15.6 (11.1–20.1)	24.5 (15.5–33.4)	19.0 (9.4–28.5)	9.6 (6.0–13.3)	21.3 (19.1–23.4)	22.0 (19.6–24.3)	17.0 (15.3–18.7)
District of Columbia	30.8 (27.4–34.2)	26.0 (22.3–29.8)	21.8 (19.2–24.5)	15.2 (8.1–22.4)	—	7.8 (3.3–12.2)	19.8 (10.7–28.9)	16.2 (7.4–25.1)	16.4 (10.8–22.0)	9.6 (7.5–11.7)	7.3 (4.9–9.6)	6.9 (5.0–8.9)
Florida	16.4 (12.9–19.9)	15.1 (11.8–18.4)	11.5 (8.4–14.6)	15.1 (12.3–17.8)	15.3 (12.5–18.1)	12.7 (9.7–15.6)	20.5 (15.9–25.1)	20.3 (14.3–26.3)	15.8 (11.1–20.5)	21.2 (19.8–22.6)	18.9 (17.5–20.3)	15.9 (14.6–17.2)
Georgia	17.5 (14.9–20.1)	14.6 (12.0–17.3)	13.9 (12.0–15.8)	12.6 (7.9–17.4)	15.6 (9.5–21.7)	11.2 (8.6–13.8)	20.6 (14.6–26.6)	15.3 (9.2–21.4)	12.1 (8.8–15.3)	24.2 (22.5–25.9)	19.3 (17.4–21.1)	18.5 (17.1–19.9)
Hawaii	—	—	—	26.8 (20.4–33.1)	23.0 (17.9–28.1)	21.4 (16.5–26.2)	15.9 (14.2–17.5)	14.4 (12.9–15.8)	13.2 (11.8–14.6)	15.4 (12.8–17.9)	10.1 (8.2–12.1)	11.6 (9.9–13.4)
Idaho	—	—	—	18.3 (11.2–25.3)	9.6 (5.6–13.6)	8.0 (5.0–11.1)	21.8 (11.2 32.3)	31.5 (21.9–41.1)	27.0 (15.9–38.2)	16.9 (15.1–18.6)	16.1 (14.5–17.8)	14.9 (13.0–16.7)
Illinois	27.1 (21.4–32.8)	24.5 (19.2–29.8)	21.4 (17.4–25.5)	23.5 (16.6–30.4)	12.9 (9.2–16.6)	10.0 (7.4–12.6)	13.5 (7.2–19.8)	8.6 (3.9–13.4)	11.0 (7.2–14.8)	19.8 (18.0–21.6)	16.3 (14.6–17.9)	16.1 (14.5–17.8)
Indiana	31.4 (26.0–36.9)	27.1 (22.3–32.0)	20.8 (16.2–25.4)	22.4 (14.8–30.0)	14.1 (9.5–18.7)	12.9 (7.9–18.0)	33.4 (24.4–42.4)	22.8 (16.6–29.0)	20.6 (13.9–27.3)	25.0 (23.6–26.5)	23.1 (21.8–24.3)	21.8 (20.4–23.2)
Iowa	32.9 (22.9–42.9)	25.3 (15.2–35.3)	22.7 (15.0–30.5)	18.9 (12.1–25.6)	17.6 (10.3–24.8)	14.2 (10.6–17.9)	31.8 (23.2–40.4)	26.6 (17.4–35.8)	25.6 (19.2–32.0)	19.9 (18.6–21.1)	18.1 (16.9–19.3)	16.1 (15.2–17.1)
Kansas	28.2 (23.6–32.9)	25.5 (20.8–30.1)	21.2 (15.6–26.9)	22.6 (19.2–26.1)	14.2 (11.3–17.0)	17.0 (12.7–21.2)	29.7 (25.0–34.4)	26.5 (21.9–31.1)	22.2 (17.1–27.3)	21.1 (20.2–21.9)	17.5 (16.6–18.4)	16.8 (15.8–17.8)
Kentucky	33.8 (26.7–40.9)	29.7 (22.5–36.9)	24.8 (17.6–31.9)	28.2 (17.1–39.2)	26.5 (14.1–38.8)	20.3 (9.7–30.9)	37.7 (26.1–49.2)	37.3 (27.3–47.4)	25.7 (16.1–35.2)	28.5 (27.0–30.0)	25.5 (24.0–27.1)	23.4 (21.7–25.1)
Louisiana	25.4 (22.6–28.2)	24.6 (22.0–27.3)	16.1 (13.4–18.7)	18.2 (11.5–24.9)	19.8 (12.1–27.6)	21.1 (12.7–29.5)	32.0 (24.0–40.1)	26.8 (19.5–34.1)	18.3 (11.9–24.6)	26.1 (24.4–27.8)	23.8 (22.2–25.5)	22.9 (20.9–24.9)
Maine	—	—	—	32.0 (17.2–46.9)	—	26.9 (11.2–42.6)	41.3 (33.3–49.3)	32.9 (25.0–40.7)	26.5 (18.2–34.8)	22.3 (21.2–23.4)	18.5 (17.3–19.8)	17.6 (16.3–18.9)
Maryland	18.9 (16.2–21.6)	16.8 (14.1–19.5)	13.7 (12.0–15.4)	19.9 (12.7–27.1)	8.2 (4.1–12.2)	6.8 (4.6–9.0)	16.8 (11.6–22.0)	9.5 (5.9–13.2)	10.7 (7.7–13.8)	19.6 (18.0–21.2)	15.5 (13.9–17.1)	13.2 (12.2–14.3)
Massachusetts	17.1 (13.6–20.6)	16.6 (12.1–21.1)	10.9 (6.8–15.1)	19.2 (15.7–22.7)	17.1 (13.2–21.1)	13.7 (10.0–17.4)	16.2 (12.6–19.7)	13.6 (10.1–17.1)	9.1 (6.0–12.2)	18.3 (17.3–19.4)	14.4 (13.3–15.5)	14.1 (12.7–15.5)
Michigan	27.1 (23.1–31.1)	22.3 (18.4–26.2)	22.7 (19.5–26.0)	20.0 (13.2–26.7)	32.4 (22.6–42.2)	25.9 (19.2–32.7)	24.5 (18.6–30.5)	26.2 (20.3–32.0)	21.8 (17.1–26.6)	22.8 (21.4–24.3)	20.1 (18.8–21.5)	17.8 (16.7–18.9)
Minnesota	29.8 (24.1–35.6)	22.3 (17.4–27.2)	21.4 (17.2–25.7)	20.1 (13.7–26.5)	14.3 (10.3–18.3)	13.6 (10.6–16.7)	25.2 (20.3–30.0)	17.7 (14.3–21.2)	18.0 (14.9–21.0)	18.2 (17.2–19.2)	15.9 (15.1–16.6)	14.6 (13.9–15.3)
Mississippi	22.8 (20.5–25.1)	20.7 (17.7–23.8)	18.8 (16.4–21.1)	25.3 (14.6–36.0)	—	—	39.8 (29.3–50.2)	—	27.6 (17.7–37.5)	27.2 (25.4–28.9)	25.3 (22.7–27.9)	21.4 (19.5–23.3)
Missouri	28.0 (23.0–33.0)	21.2 (16.5–26.0)	22.1 (16.8–27.4)	16.2 (8.0–24.4)	—	18.1 (8.9–27.4)	27.7 (19.0–36.4)	13.8 (8.5–19.1)	24.1 (16.5–31.8)	24.9(23.2–26.6)	20.8 (19.2–22.4)	19.0 (17.3–20.6)
Montana	—	—	—	40.1 (27.5–52.6)	32.2 (19.1–45.3)	20.6 (8.7–32.6)	40.9 (35.9–45.9)	38.6 (32.6–44.6)	36.3 (30.3–42.3)	19.9 (18.6–21.2)	17.8 (16.3–19.3)	16.5 (14.9–18.0)
Nebraska	28.0 (23.1–32.9)	19.9 (14.2–25.6)	29.3 (22.2–36.3)	18.4 (15.3–21.5)	14.0 (10.6–17.4)	12.2 (9.3–15.0)	24.4 (20.0–28.7)	27.3 (21.3–33.2)	22.2 (16.4–28.0)	19.5(18.7–20.3)	17.0 (16.1–17.9)	15.3 (14.3–16.3)
Nevada	30.0 (21.8–38.2)	24.6 (15.6–33.5)	21.6 (13.4–29.9)	15.5 (11.2–19.9)	15.8 (11.3–20.4)	11.2 (7.8–14.6)	21.1 (14.6–27.5)	14.7 (7.9–21.4)	16.7 (10.4–23.1)	25.7 (23.2–28.1)	17.2 (14.9–19.6)	16.6 (14.3–18.9)
New Hampshire	—	—	—	25.5 (11.8–39.3)	—	—	33.3 (24.2–42.4)	24.6 (14.9–34.4)	23.7 (14.1–33.4)	19.0(17.5–20.4)	17.3 (15.7–18.8)	15.6 (14.1–17.2)
New Jersey	21.2 (18.2–24.2)	18.0 (15.1–20.9)	12.7 (6.5–19.0)	14.5 (12.1–16.8)	14.2 (11.7–16.7)	13.4 (9.1–17.7)	10.2 (7.4–12.9)	10.4 (7.5–13.3)	—	17.8 (16.6–19.0)	15.9 (14.6–17.2)	14.4 (11.5–17.3)
New Mexico	32.7 (20.4–45.1)	—	—	23.0 (21.0–25.0)	20.0 (17.7–22.3)	15.6 (13.5–17.6)	16.9 (13.4–20.3)	13.7 (10.0–17.3)	16.5 (13.1–20.0)	20.8 (19.1–22.4)	19.2 (17.1–21.2)	14.7 (13.1–16.4)
New York	21.3 (17.5–25.0)	16.1 (12.7–19.5)	14.1 (12.2–16.1)	17.4 (14.0–20.7)	14.1 (11.0–17.2)	12.1 (10.4–13.9)	17.2 (12.8–21.6)	8.6 (5.8–11.4)	9.2 (7.3–11.1)	17.9 (16.4–19.5)	15.0 (13.6–16.5)	13.3 (12.5–14.1)
North Carolina	23.4 (20.2–26.6)	20.6 (17.9–23.3)	17.2 (14.0–20.4)	16.6 (11.5–21.8)	11.7 (8.6–14.8)	10.8 (7.0–14.6)	23.9 (18.3–29.5)	18.8 (13.4–24.3)	18.0 (11.8–24.2)	21.8(20.2–23.3)	19.4 (18.0–20.9)	18.3 (16.5–20.1)
North Dakota	—	—	19.8 (9.2–30.3)	—	51.0 (32.9–69.1)	25.3 (12.3–38.2)	48.4 (39.1–57.6)	40.2 (31.4–49.0)	38.1 (30.3–45.9)	20.2(18.6–21.7)	17.6 (16.1–19.1)	17.5 (15.9–19.1)
Ohio	27.2 (22.9–31.5)	22.0 (17.3–26.7)	23.4 (19.2–27.7)	19.2 (10.4–27.9)	20.8 (11.9–29.6)	18.6 (12.2–24.9)	36.5 (27.2–45.8)	22.6 (14.9–30.3)	23.8 (16.2–31.5)	24.6 (23.1–26.0)	20.7 (19.3–22.1)	20.1 (18.9–21.3)
Oklahoma	30.7 (24.5–36.9)	25.6 (20.3–30.8)	21.0 (15.0–27.1)	18.1 (12.8–23.5)	13.3 (9.1–17.5)	11.6 (7.4–15.8)	28.4 (24.6–32.2)	28.1 (24.2–32.0)	24.2 (19.9–28.5)	26.0 (24.4–27.6)	20.2 (18.8–21.6)	19.8 (18.1–21.6)
Oregon	—	—	—	19.7 (12.6–26.8)	12.7 (7.9–17.6)	12.1 (8.8–15.3)	18.0 (13.0–23.0)	20.1 (14.0–26.2)	18.4 (12.9–23.8)	20.0 (18.4–21.5)	17.1 (15.5–18.7)	16.1 (14.7–17.5)
Pennsylvania	28.7 (24.5–32.9)	24.1 (19.8–28.3)	23.7 (19.3–28.0)	27.1 (20.6–33.7)	25.1 (17.7–32.6)	18.9 (13.2–24.6)	27.5 (21.0–34.0)	13.2 (8.3–18.0)	14.7 (10.3–19.1)	21.2(20.0–22.4)	19.4 (18.2–20.6)	16.3 (14.9–17.7)
Rhode Island	21.8 (13.4–30.3)	21.9 (13.8–30.1)	9.5 (4.3–14.7)	19.8 (14.8–24.9)	13.6 (8.9–18.4)	9.7 (6.1–13.4)	21.8 (15.6–28.0)	17.4 (9.2–25.5)	17.1 (11.1–23.2)	19.9 (18.3–21.5)	16.2 (14.6–17.8)	15.5 (13.7–17.3)
South Carolina	23.5 (21.1–25.9)	21.9 (19.5–24.3)	17.0 (14.7–19.3)	18.5 (11.5–25.4)	20.6 (13.8–27.4)	12.8 (8.0–17.7)	25.8 (16.3–35.4)	31.1 (24.7–37.5)	21.5 (15.0–27.9)	23.2 (21.7–24.7)	20.9 (19.5–22.3)	18.7 (17.3–20.1)
South Dakota	—	—	—	23.5 (11.4–35.6)	—	30.6 (16.6–44.5)	39.6 (32.2–47.1)	34.7 (28.4–41.0)	31.2 (24.5–38.0)	21.1 (19.0–23.1)	16.7 (15.1–18.4)	16.9 (15.0–18.8)
Tennessee	18.7 (12.4–25.0)	21.5 (16.3–26.7)	20.8 (16.1–25.4)	—	—	9.3 (5.0–13.6)	36.7 (20.0–53.4)	26.9 (17.1–36.8)	23.4 (16.5–30.4)	23.2 (20.6–25.8)	24.7 (22.6–26.7)	21.0 (19.3–22.8)
Texas	25.0(20.4–29.6)	13.9 (10.7–17.1)	21.0 (16.0–26.0)	15.7 (13.7–17.7)	13.4 (11.7–15.0)	12.0 (9.3–14.7)	14.2 (10.2–18.2)	10.6 (6.9–14.3)	13.1 (8.2–18.1)	21.0 (19.3–22.6)	16.1 (14.7–17.5)	14.6 (12.7–16.6)
Utah	29.1 (14.8–43.4)	—	15.5 (5.0–26.1)	14.5 (11.4–17.7)	10.4 (8.3–12.6)	11.8 (9.2–14.4)	14.7 (10.3–19.0)	11.5 (8.2–14.8)	14.1 (10.3–17.8)	11.0 (10.2–11.9)	9.3 (8.7–10.0)	8.1 (7.4–8.9)
Vermont	—	—	—	—	8.0 (0.0–18.0)	—	37.8 (27.5–48.2)	31.2 (23.1–39.2)	29.6 (19.5–39.8)	18.2 (16.7–19.6)	15.9 (14.7–17.1)	12.9 (11.7–14.2)
Virginia	23.1 (19.0–27.2)	19.5 (16.5–22.4)	18.1 (15.2–20.9)	17.8 (12.0–23.6)	15.2 (10.5–19.9)	8.4 (5.7–11.0)	22.1 (15.6–28.7)	19.8 (15.2–24.4)	10.5 (6.9–14.1)	20.5 (18.7–22.2)	20.2 (18.9–21.6)	15.6 (14.4–16.7)
Washington	25.7 (16.7–34.8)	17.4 (9.5–25.4)	12.0 (7.2–16.9)	13.9 (10.0–17.8)	13.3 (9.5–17.1)	10.2 (7.8–12.7)	17.0 (13.4–20.5)	17.0 (13.2–20.7)	13.1 (10.3–15.8)	17.7 (16.5–19.0)	15.3 (14.1–16.4)	12.3 (11.4–13.3)
West Virginia	34.3 (23.4–45.1)	25.7 (16.5–34.8)	27.4 (16.6–38.1)	35.9 (19.0–52.8)	—	—	28.0 (17.2–38.8)	26.1 (16.7–35.6)	28.2 (18.4–37.9)	28.4 (26.7–30.0)	26.7 (25.3–28.2)	25.1 (23.4–26.7)
Wisconsin	37.5 (27.8–47.1)	35.1 (23.5–46.8)	17.1 (9.6–24.7)	27.4 (12.4–42.4)	15.3 (8.9–21.7)	17.4 (10.2–24.5)	19.6 (9.1–30.1)	20.0 (13.8–26.3)	16.2 (9.7–22.7)	19.8 (18.0–21.5)	16.4 (15.0–17.8)	16.4 (14.9–18.0)
Wyoming	—	—	—	26.8 (19.8–33.8)	—	26.6 (20.0–33.2)	33.5 (24.3–42.7)	37.0 (25.1–48.8)	32.0 (22.2–41.9)	22.1 (20.5–23.7)	18.8 (16.9–20.7)	17.4 (15.8–19.0)

a All racial/ethnic groups are non-Hispanic except for the Hispanic group. Estimates were suppressed if the relative standard error was greater than 30% or the denominator of the estimate was less than 50. Dashes indicate that the estimate was suppressed.

### Time trends in smoking across racial/ethnic groups, 2011–2018

In all states except Tennessee, the odds of smoking significantly decreased from 2011 to 2018 (odds ratio [OR] range, 0.94–0.98), after adjusting for age, sex, race/ethnicity, and education level (Table 3). Significant declines in the odds of smoking were estimated using a linear time trend in most states. However, in 9 states (Hawaii, Illinois, Indiana, Kansas, Massachusetts, South Dakota, Texas, Utah, Wyoming) a quadratic time trend was significant. In these 9 states with a quadratic time trend, the odds of smoking decreased and accelerated from 2011 to 2018, indicating that the odds of smoking declined from 2011 to 2018 and at a faster rate over time. In Tennessee, there was no significant change in the odds of smoking from 2011 to 2018 (OR, 0.98 [95% CI, 0.97–1.00]).

**Table 3:** Odds Ratios of the Race/Ethnicity and Year Terms in the Logistic Regression Models, Behavioral Risk Factor Surveillance System, 2011–2018^a^

State	Black		Hispanic		Other		Year		Year-Squared	
	OR (95% CI)	*P*	OR (95% CI)	*P*	OR (95% CI)	*P*	OR (95% CI)	*P*	OR (95% CI)	*P*
Alabama	0.74 (0.69–0.80)	<.001	—	—	1.21 (1.02–1.43)	.027	0.97 (0.96–0.99)	<.001	—	—
Alaska	—	—	—	—	1.95 (1.75–2.17)	<.001	0.98 (0.96–1.00)	.04	—	—
Arizona	—	—	0.57 (0.51–0.64)	<.001	0.80 (0.69–0.92)	.002	0.96 (0.94–0.98)	<.001	—	—
Arkansas	0.82 (0.72–0.93)	.002	—		1.24 (1.02–1.51)	.03	0.97 (0.96–0.99)	.004	—	—
California	1.11 (0.99–1.25)	.08	0.55 (0.51–0.59)	<.001	0.74 (0.67–0.81)	<.001	0.98 (0.96–0.99)	<.001	—	—
Colorado	1.29 (1.12–1.48)	<.001	0.78 (0.73–0.84)	<.001	1.09 (0.97–1.23)	.15	0.96 (0.95–0.98)	<.001	—	—
Connecticut	1.01 (0.90–1.13)	.85	0.84 (0.76–0.93)	.001	0.87 (0.75–1.02)	.08	0.95 (0.94–0.96)	<.001	—	—
Delaware	0.78 (0.70–0.88)	<.001	0.47 (0.39–0.56)	<.001	0.87 (0.72–1.05)	.14	0.96 (0.95–0.98)	<.001	—	—
District of Columbia	1.98 (1.69–2.30)	<.001	—	—	1.44 (1.14–1.82)	.002	0.94 (0.92–0.96)	<.001	—	—
Florida	0.58 (0.52–0.64)	<.001	0.55 (0.50–0.60)	<.001	0.85 (0.74–0.97)	.01	0.97 (0.96–0.98)	<.001	—	—
Georgia	0.66 (0.61–0.72)	<.001	—	—	0.79 (0.67–0.93)	.004	0.97 (0.95–0.98)	<.001	—	—
Hawaii	—	—	1.34 (1.16–1.54)	<.001	1.03 (0.95–1.13)	.48	0.90 (0.83–0.97)	.004	1.01 (1.00–1.02)	.02
Idaho	—	—	0.56 (0.46–0.67)	<.001	1.90 (1.56–2.31)	<.001	0.97 (0.95–0.99)	.007	—	—
Illinois	1.12 (1.00–1.26)	.046	0.47 (0.40–0.54)	<.001	0.80 (0.66–0.95)	.01	0.87 (0.80–0.93)	<.001	1.01 (1.00–1.02)	.004
Indiana	0.95 (0.86–1.06)	.37	0.45 (0.38–0.52)	<.001	1.13 (0.98–1.31)	.10	0.90 (0.85–0.95)	<.001	1.01 (1.00–1.02)	.004
Iowa	—	—	—	—	1.53 (1.29–1.82)	<.001	0.97 (0.96–0.99)	<.001	—	—
Kansas	1.25 (1.14–1.38)	<.001	0.58 (0.53–0.64)	<.001	1.29 (1.18–1.42)	<.001	0.90 (0.86–0.94)	<.001	1.01 (1.00–1.01)	<.001
Kentucky	0.90 (0.79–1.01)	.08	—	—	1.36 (1.15–1.61)	<.001	0.97 (0.96–0.98)	<.001	—	—
Louisiana	0.73 (0.67–0.79)	<.001	—	—	0.96 (0.80–1.15)	.67	0.97 (0.96–0.99)	<.001	—	—
Maine	—	—	—	—	1.55 (1.34–1.79)	<.001	0.97 (0.96–0.98)	<.001	—	—
Maryland	0.80 (0.74–0.86)	<.001	—	—	0.72 (0.62–0.83)	<.001	0.95 (0.94–0.96)	<.001	—	—
Massachusetts	0.79 (0.69–0.91)	.001	0.64 (0.57–0.72)	<.001	0.88 (0.78–1.00)	.05	0.90 (0.85–0.96)	<.001	1.01 (1.00–1.01)	.04
Michigan	1.10 (1.01–1.19)	.02	0.89 (0.76–1.04)	.14	1.29 (1.16–1.45)	<.001	0.97 (0.96–0.98)	<.001	—	—
Minnesota	1.18 (1.05–1.32)	.004	—	—	1.10 (0.99–1.21)	.08	0.96 (0.95–0.97)	<.001	—	—
Mississippi	0.69 (0.64–0.75)	<.001	—	—	—	—	0.97 (0.96–0.98)	<.001	—	—
Missouri	—	—	—	—	1.19 (1.02–1.39)	.03	0.97 (0.96–0.99)	<.001	—	—
Montana	—	—	—	—	2.29 (2.08–2.53)	<.001	0.97 (0.96–0.99)	<.001	—	—
Nebraska	1.25 (1.09–1.43)	.001	0.55 (0.49–0.60)	<.001	1.33 (1.17–1.50)	<.001	0.96 (0.95–0.97)	<.001	—	—
Nevada	1.16 (0.97–1.39)	.09	0.53 (0.46–0.61)	<.001	0.93 (0.78–1.11)	.41	0.96 (0.94–0.98)	<.001	—	—
New Hampshire	—	—	—	—	1.47 (1.22–1.77)	<.001	0.98 (0.97–1.00)	.038	—	—
New Jersey	0.96 (0.87–1.07)	.48	0.55 (0.50–0.62)	<.001	—	—	0.96 (0.95–0.98)	<.001	—	—
New Mexico	—	—	0.77 (0.72–0.83)	<.001	0.62 (0.55–0.70)	<.001	0.96 (0.95–0.97)	<.001	—	—
New York	0.83 (0.76–0.92)	<.001	0.59 (0.54–0.65)	<.001	0.63 (0.56–0.72)	<.001	0.96 (0.95–0.97)	<.001	—	—
North Carolina	0.91 (0.84–0.98)	.01	0.33 (0.29–0.38)	<.001	0.87 (0.76–1.00)	.04	0.97 (0.96–0.98)	<.001	—	—
North Dakota	—	—	—	—	2.44 (2.14–2.78)	<.001	0.97 (0.96–0.99)	<.001	—	—
Ohio	1.04 (0.95–1.13)	.45	0.84 (0.70–1.01)	.07	1.23 (1.06–1.42)	.006	0.98 (0.97–0.99)	<.001	—	—
Oklahoma	0.98 (0.87–1.11)	.74	0.54 (0.47–0.62)	<.001	1.22 (1.12–1.33)	<.001	0.96 (0.94–0.97)	<.001	—	—
Oregon	1.20 (0.88–1.63)	.26	—	—	1.06 (0.93–1.22)	.39	0.98 (0.96–0.99)	.002	—	—
Pennsylvania	1.22 (1.11–1.34)	<.001	0.96 (0.83–1.10)	.55	1.01 (0.87–1.18)	.89	0.96 (0.95–0.98)	<.001	—	—
Rhode Island	0.82 (0.68–0.98)	.03	0.50 (0.43–0.58)	<.001	1.05 (0.89–1.25)	.57	0.96 (0.94–0.97)	<.001	—	—
South Carolina	0.79 (0.74–0.84)	<.001	0.51 (0.43–0.61)	<.001	1.14 (0.99–1.32)	.07	0.96 (0.95–0.98)	<.001	—	—
South Dakota	—	—	—	—	2.29 (2.04–2.59)	<.001	0.87 (0.80–0.95)	.001	1.01 (1.00–1.02)	.01
Tennessee	0.77 (0.69–0.87)	<.001	—	—	1.14 (0.92–1.41)	.24	0.99 (0.97–1.00)	.09	—	—
Texas	0.89 (0.79–1.00)	.05	0.55 (0.51–0.60)	<.001	0.80 (0.69–0.93)	.004	0.86 (0.80–0.92)	<.001	1.01 (1.00–1.02)	.001
Utah	—	—	0.93 (0.84–1.03)	.15	1.44 (1.25–1.66)	<.001	0.89 (0.84–0.94)	<.001	1.01 (1.00–1.02)	.009
Vermont	—	—	—	—	1.88 (1.59–2.22)	<.001	0.97 (0.96–0.99)	<.001	—	—
Virginia	0.92 (0.85–1.00)	.05	0.52 (0.45–0.60)	<.001	0.86 (0.75–0.98)	.02	0.95 (0.94–0.96)	<.001	—	—
Washington	0.98 (0.83–1.16)	.83	0.54 (0.48–0.60)	<.001	0.99 (0.91–1.09)	.87	0.95 (0.94–0.96)	<.001	—	—
West Virginia	1.17 (0.98–1.40)	.08	—	—	1.27 (1.07–1.51)	.007	0.98 (0.97–1.00)	<.001	—	—
Wisconsin	1.45 (1.21–1.74)	<.001	—	—	1.16 (0.96–1.40)	.12	0.96 (0.95–0.98)	.01	—	—
Wyoming	—	—	—	—	1.58 (1.30–1.91)	<.001	0.88 (0.82–0.95)	.002	1.01 (1.00–1.02)	.02

Abbreviation: OR, odds ratio.

a Logistic regression models were adjusted for age, sex, and education; the reference group is non-Hispanic White. Models were run separately for each state. The year variable refers to the linear time trend. The year-squared variable refers to the quadratic time trend. The quadratic time trend was dropped from the logistic regression model if it was not significant (*P* < .05). All racial/ethnic groups are non-Hispanic except for the Hispanic group. Estimates were suppressed if the relative standard error was greater than 30% or the denominator of the estimate was less than 50. Dashes indicate that the estimate was suppressed.

Among 36 states with reliable estimates for smoking prevalence among Black and White adults, from 2011 to 2018, compared with White adults, the odds of smoking were lower among Black adults in 14 states (OR range, 0.58 [95% CI, 0.52–0.64] to 0.91 [95% CI, 0.84–0.98]), higher in 9 states (OR range, 1.10 [95% CI, 1.01–1.19] to 1.98 [95% CI, 1.69–2.30]), and there were no significant differences in the odds of smoking in 13 states. Among the 28 states with reliable estimates for smoking prevalence among Hispanic and White adults, compared with White adults, the odds of smoking were lower among Hispanic adults in 23 states (OR range, 0.33 [95% CI, 0.29–0.38] to 0.84 [95% CI, 0.76–0.93]) and higher in 1 state (OR, 1.34 [95% CI, 1.16–1.54]), and no significant differences were found in the odds of smoking in 4 states. Among the 49 states with reliable estimates for smoking prevalence among Other and White adults, compared with White adults, the odds of smoking were lower among Other adults in 11 states (OR range, 0.62 [95% CI, 0.55–0.70] to 0.87 [95% CI, 0.76–1.00]) and higher in 22 states (OR range, 1.19 [95% CI, 1.02–1.39] to 2.44 [95% CI, 2.14–2.78]), and no significant differences were found in 16 states.

### Differential time trends in smoking

We found significant (*P* < .05) interactions between year and race/ethnicity in 4 states, indicating that time trends varied across racial/ethnic groups (Figure) (more information available at https://tarheels.live/pcdsupplementalfile/). In 2 states (Indiana and Wisconsin), differential time trends in smoking prevalence were found across Black and White adults. In Wisconsin, simple slopes analyses indicated a significant negative effect for year among Black (OR = 0.88 [95% CI, 0.82–0.95]) and White adults (0.98 [95% CI, 0.96–1.00]), but the ORs for year were lower among Black adults. In Indiana, among Black adults, a significant negative effect for year was found from 2014–2016 (0.93 [95% CI, 0.89–0.97] to 0.94 [95% CI, 0.88–0.99]), but no significant effect for year was found from 2011–2013 or from 2017–2018. Among White adults, a significant negative effect for year was found from 2011–2014 (0.92 [95% CI, 0.87–0.97] to 0.97 [95% CI, 0.95–0.99]), but no significant effect for year was found from 2015–2018.

**Figure:**
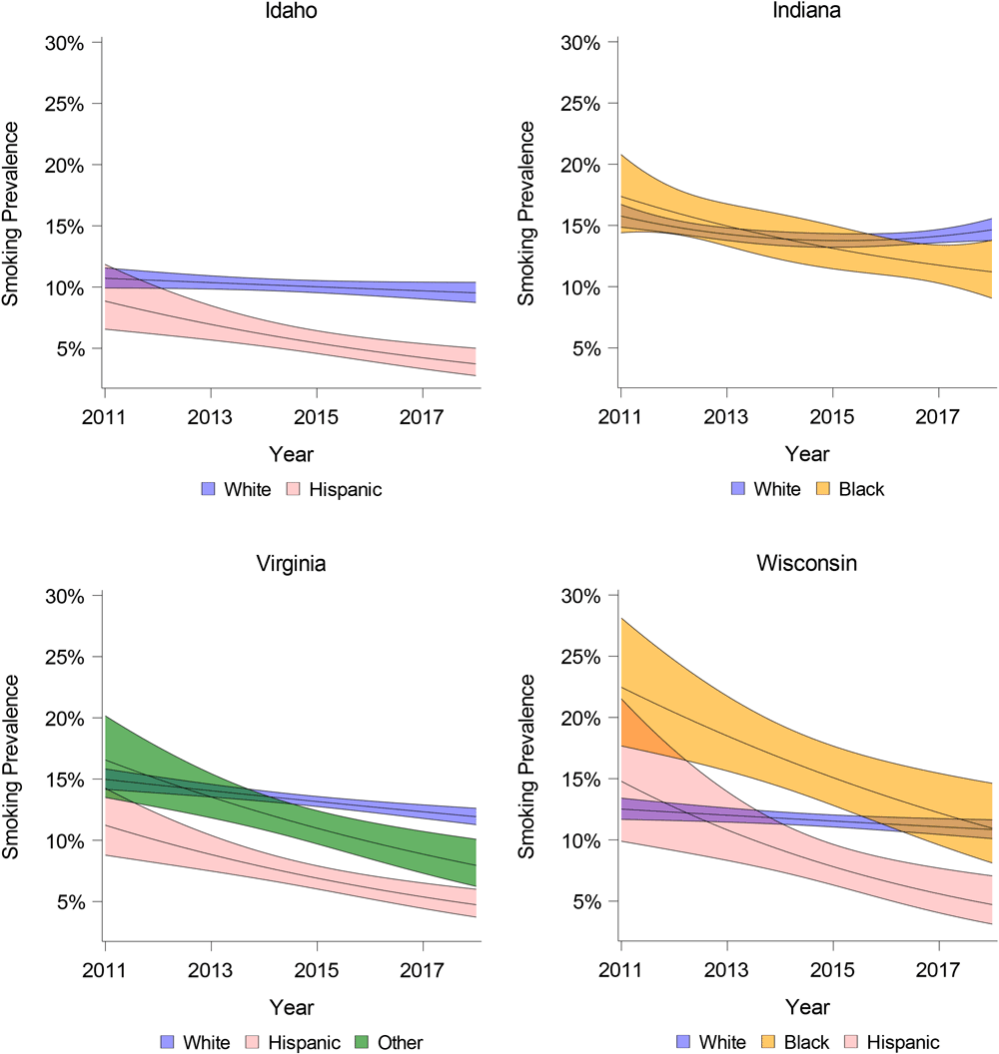
Smoking prevalence over time in Idaho, Indiana, Virginia, and Wisconsin, by race/ethnicity, Behavioral Risk Factor Surveillance System, 2011–2018. Shading indicates 95% CIs.

In 3 states (Idaho, Virginia, Wisconsin), differential time trends in smoking prevalence were found across Hispanic and White adults. In Virginia and Wisconsin, simple slopes analyses indicated a significant negative effect for year among Hispanic adults (Virginia, 0.88 [95% CI, 0.82–0.93]; Wisconsin, 0.84 [95% CI, 0.75–0.93]) and White adults (Virginia, 0.96 [95% CI, 0.95–0.98]; Wisconsin, 0.98 [95% CI, 0.96–1.00]). However, the ORs for year were lower among Hispanic adults. In Idaho, a negative effect for year was found among Hispanic adults (OR, 0.88 [95% CI, 0.81–0.95]), but no significant effect for year was found among White adults (OR, 0.98 [0.96–1.00]).

In Virginia, differential time trends in smoking prevalence were found across Other and White adults. Simple slopes analyses indicated a significant negative effect for year among Other adults (0.89 [95% CI, 0.83–0.94]) and White adults (0.96 [95% CI, 0.95–0.98]), but the effect for year was greater in Other adults.

## Discussion

Our findings suggest that national data on smoking prevalence across racial/ethnic groups may obscure important differences across states. From 2011 to 2018, the odds of smoking among Black adults were lower, not significantly different, or higher, depending on the state. The odds of smoking were lower among Hispanic adults in most states, and the odds of smoking were higher among Other adults compared with White adults in about half of states. In most other states, no significant differences were found in the odds of smoking between Other and White adults.

In all states except Tennessee, the odds of smoking declined from 2011 to 2018. In addition, in most states, trends in the odds of smoking did not vary across racial/ethnic groups over time, suggesting no change in racial/ethnic differences in smoking. In 4 states, however, time trends varied across racial/ethnic groups. In states with differential time trends, the decline in the odds of smoking was typically greater among Black, Hispanic, or Other adults compared with White adults. In Idaho, we found a significant decline in the odds of smoking among Hispanics but no significant decline in the odds of smoking among White adults.

In states with differential time trends in smoking, racial/ethnic minority groups experienced a steeper decline in the odds of smoking compared with White adults, and this resulted in similar or lower smoking prevalence among racial/ethnic minorities compared with White adults by 2018. Two states (Indiana and Wisconsin) had differential time trends in smoking between Black and White adults. In Indiana, Black adults had a similar smoking prevalence to White adults in 2011, and by the of the study period in 2018, smoking prevalence was lower among Black adults. In Wisconsin, Black adults had higher smoking prevalence than White adults in 2011, but there were no differences in smoking prevalence by the end of the study period. In 1 state (Virginia) there was a differential time trend in smoking between Other and White adults. In Virginia, Other adults had similar smoking prevalence to White adults at the start of the study period but lower smoking prevalence than White adults by 2018. Three states (Idaho, Virginia, Wisconsin) had differential time trends in smoking when comparing Hispanic and White adults. In these states, Hispanic adults had similar smoking prevalence to White adults at the start of the study period, but by 2018 smoking prevalence was lower among Hispanic adults.

State tobacco control programs should consider the role their policies play in maintaining racial/ethnic disparities in smoking. Research on the impact of tobacco control policies on racial/ethnic disparities in smoking is limited. Most research on the equity impact of tobacco control policies has focused on socioeconomic disparities in smoking (12,13). Although our study did not examine the impact of state tobacco control policies, a discussion of the tobacco control policy environment in the 4 states where racial/ethnic minority groups experienced steeper declines in smoking compared with White adults (Idaho, Indiana, Virginia, Wisconsin) may provide insights into interventions that promote equity. Compared with other US states, Wisconsin has one of the higher state excise taxes on cigarettes, and its excise tax increased by $0.75 in 2009, 2 years before the start of the study period (14). Research suggests that increasing the price of tobacco products may reduce racial/ethnic disparities in smoking (12). However, Idaho, Indiana, and Virginia, where there were also steeper declines in smoking prevalence among racial/ethnic minority groups, have some of the lowest cigarette excise taxes and did not raise taxes during the study period or in the several years prior (14). Across the study period, state-level smoke-free air laws were comprehensive in Idaho and Wisconsin but not in Indiana or Virginia (16). In addition, in each of these 4 states, state-level tobacco control program funding was below levels recommended by the Centers for Disease Control and Prevention during the study period, and access to cessation services was consistently rated as poor by the American Lung Association (16). Poor overall state-level tobacco control programs and policies in these states and no substantive change in state-level tobacco control policies over the study period suggests that other tobacco control policies and programs, such as those at the local level, or other policies that are not directed toward reducing smoking (eg, education-related policies), may be in part responsible for the steeper declines in smoking among racial/ethnic minority groups. Studies that examine the impact of policies on racial/ethnic disparities in smoking are needed to guide policy makers and tobacco control programs. In addition, trends in smoking prevalence across racial/ethnic groups should be consistently monitored to identify groups for which progress is not being made. Ideally, after controlling for factors associated with smoking such as age, sex, and education, no differences should be found in smoking prevalence across racial/ethnic groups.

Our study has limitations. This study was descriptive and did not examine the impact of state tobacco control programs or policies, and it was limited to states with reliable smoking estimates. In several states, the smoking estimate for certain racial/ethnic groups was not reliable, and cross-sectional estimates and trends in smoking prevalence in those states could not be examined. In addition, because of small sample sizes, adults who were not Black, White, or Hispanic were combined into a single racial/ethnic group. State tobacco control programs should consider data collection that oversamples racial/ethnic groups with smaller population sizes in their states so reliable smoking estimates for all population groups can be obtained. Our study did not control for the false discovery rate or for potential type I error due to multiple testing because it was exploratory, and we had a greater concern of avoiding type II error. However, *P *values for all time trends were presented, so adjustment can be made if desired.

In summary, racial/ethnic disparities in smoking prevalence varied across US states. In addition, in most states, trends in the odds of smoking across racial/ethnic groups remained stable over time. In some states, the odds of smoking declined more quickly among racial/ethnic minority adults than among White adults, suggesting that some progress has been made in reducing racial/ethnic disparities in smoking. However, additional efforts are needed to eliminate racial/ethnic disparities in smoking.

## Post-Test Information

To obtain credit, you should first read the journal article. After reading the article, you should be able to answer the following, related, multiple-choice questions. To complete the questions (with a minimum 75% passing score) and earn continuing medical education (CME) credit, please go to http://www.medscape.org/journal/pcd. Credit cannot be obtained for tests completed on paper, although you may use the worksheet below to keep a record of your answers.

You must be a registered user on http://www.medscape.org. If you are not registered on http://www.medscape.org, please click on the “Register” link on the right hand side of the website.

Only one answer is correct for each question. Once you successfully answer all post-test questions, you will be able to view and/or print your certificate. For questions regarding this activity, contact the accredited provider, CME@medscape.net. For technical assistance, contact CME@medscape.net. American Medical Association’s Physician’s Recognition Award (AMA PRA) credits are accepted in the US as evidence of participation in CME activities. For further information on this award, please go to https://www.ama-assn.org. The AMA has determined that physicians not licensed in the US who participate in this CME activity are eligible for AMA PRA Category 1 Credits™. Through agreements that the AMA has made with agencies in some countries, AMA PRA credit may be acceptable as evidence of participation in CME activities. If you are not licensed in the US, please complete the questions online, print the AMA PRA CME credit certificate, and present it to your national medical association for review.

## Post-Test Questions

### Study Title: State-Level Patterns and Trends in Cigarette Smoking Across Racial and Ethnic Groups in the United States, 2011–2018

#### CME Questions

1. Which of the following statements regarding the prevalence of cigarette smoking in the United States in 2018 is most accurate?
  - The overall rate of smoking among US adults was 4.1%
  - The overall rates of smoking among US adults was 24.9%
  - Smoking rates were higher among Hispanic vs White adults
  - Smoking rates were higher among Black vs Hispanic adults
2. Which of the following statements regarding rates of smoking among Black adults between 2011 and 2018 is most accurate?
  - Rates of smoking were higher among Black vs White adults in nearly all states
  - There were more states in which Black adults had lower smoking rates compared with White adults vs states in which the rate was higher among Black adults
  - There was a difference in smoking rates in comparing Black and White adults in just 2 states
  - Rates of smoking were higher among White vs Black adults in nearly all states
3. Which of the following statements regarding rates of smoking among Hispanic adults between 2011 and 2018 is most accurate?
  - Rates of smoking were higher among Hispanic vs White adults in the vast majority of states
  - Rates of smoking were largely similar in comparing Hispanic and White adults across different states
  - There was a difference in smoking rates in comparing Hispanic and White adults in just 2 states
  - Rates of smoking were higher among White vs Hispanic adults in the vast majority of states
4. Which of the following statements regarding temporal trends in smoking between 2011 and 2018 in the current study is most accurate?
  - The prevalence of smoking declined in 60% of states during the study period
  - There were no differences in smoking trends according to race/ethnicity in most states during the study period
  - The reduction in the rate of smoking among Black adults was less pronounced during the study period
  - The reduction in the rate of smoking among Hispanic adults was less pronounced during the study period
